# Examining the association of social needs with dental utilization

**DOI:** 10.3389/froh.2026.1884784

**Published:** 2026-07-10

**Authors:** David M. Mosen, Matthew P. Banegas, Alex M. Varga, Daniel J. Pihlstrom, Briar L. Ertz-Berger, John F. Dickerson

**Affiliations:** 1 Kaiser Permanente Center for Health Research, Portland, OR, United States; 2 Radiation Medicine and Applied Sciences, University of California, San Diego, La Jolla, CA, United States; 3 Permanente Dental Group (PDG), Portland, OR, United States; 4 Continuum of Care Department, Northwest Permanente, Portland, OR, United States

**Keywords:** dental utilization, insurance coverage, medical dental integration, social determinants of health, social needs

## Abstract

**Introduction:**

The objective of this study was to: 1) describe the prevalence of social needs in an integrated health system in Oregon and Southwest Washington and 2) examine whether and how the presence of social needs is associated with dental care utilization.

**Methods:**

In this observational study, we identified 80,273 patients that met the following inclusion criteria: 1) completed a social needs screening between 1/1/2023 and 10/1/2024 (index date), 2) age 18+ as of index date, 3) 12+ months of dental and medical insurance coverage before and after index date and 4) not on research exclusion list. Four social needs were assessed: financial strain, food insecurity, housing instability and transportation needs. Two logistic regression models analyzed the association of social needs with dental care utilization: 1) each social need variable analyzed as a dichotomous measure: yes vs. no (ref. group) and 2) social needs analyzed as a count variable: 1, 2, 3 or more vs. 0 needs (ref. group).

**Results:**

Approximately 17.8% of patients reported ≥1 social need at index date. We found that those with financial strain, food insecurity and transportation needs had lower odds of having any dental visits in the 12 months after social needs assessment. Having 1 or more social needs was associated with lower odds of having any dental visit.

**Discussion:**

Further research is needed to evaluate the effectiveness of interventions to target social needs and whether resolution of these needs is associated with increases in dental utilization.

## 1 Introduction

The presence of social needs, such as financial strain and housing instability, has been associated with worse health outcomes (1–3). Similarly, social needs have been linked to poor access to medical care (4) and lower overall use of primary care medical visits (5, 6). However, less is known about how social needs impact routine dental utilization. This area of research is important, since regular access to dental area is linked to not only improved oral health outcomes (7) but also improved physical health outcomes (8, 9).

Among social needs, financial strain or difficulty paying for dental care has been studied most extensively. Several studies found difficulty paying for dental care remains a large barrier to accessing dental care (10–12). Research indicates that more people reported financial barriers to receiving dental care, compared to any other type of health care (10); fewer than 30% of adults ≥65 have dental insurance and 8% are unable to afford dental care (13).

Other research has found that food insecurity (14–16), housing instability (15, 17) and transportation needs (15, 18, 19) negatively impact dental utilization. However, few studies have examined the association of social needs with dental utilization in integrated health systems. Examining the associations of social needs with dental care utilization in integrated health systems can: 1) provide learnings from systems with comprehensive data on social needs, demographic characteristics, and health care utilization and 2) identify possible interventions for populations with specific social needs. Thus, the objective of our study is to: 1) describe the prevalence of social needs in Kaiser Permanente Northwest (KPNW), an integrated health system in Oregon and Southwest Washington and 2) examine whether and how the presence of social needs is associated with dental care utilization. KPNW provides a unique opportunity to conduct research with its integrated medical and dental health record as demonstrated by previous research examining the association of medical/dental integration with care gap closure (20, 21).

## 2 Methods

This retrospective, observational study used data from adult members of KPNW, an integrated health care system in Oregon and Southwest Washington. To be included, KPNW members met the following criteria: 1) completed an Epic-based social needs assessment between 1/1/2023 and 10/1/2024 (index date; KPNW members complete assessments as part of routine healthcare), 2) age 18 or older upon index date, 3) ≥12 months of KPNW medical and dental insurance coverage before and ≥12 months of medical and dental insurance coverage after index date and 4) not on the KPNW research exclusion list. The study was approved by the Kaiser Permanente Northwest Institutional Review Board. The Epic-based social needs screener is based on previously validated questions published in the research literature (22–29).

### 2.1 Measures

#### 2.1.1 Outcome measure

The outcome measure for the study was any use of dental care (yes vs. no), measured 12 months after the index date. Dental utilization data was obtained from KPNW's electronic health record (EHR).

#### 2.1.2 Independent variables

The presence of social needs, also identified from the KPNW EHR, was measured in two ways. First, four social needs measures (financial strain, food insecurity, housing instability, transportation needs) assessed on the index date were categorized as binary measures (yes vs. no). These measures have been reported in previous publications (30, 31) and are reported in Appendix A. Second, the total count of social needs was analyzed as: none, one social need, two social needs, and three or more social needs.

#### 2.1.3 Covariate measures

Covariate measures were identified from KPNW's EHR. These measures included three demographic measures: age, sex, and race/ethnicity; one socioeconomic measure, the Area Deprivation Index (ADI) (32) and one clinical comorbidity measure, the Charlson Comorbidity Index (CCI) (33, 34). Further, four utilization measures were assessed in the 12 months prior to the social needs assessment date: dental utilization, primary care utilization, emergency department (ED) visits and hospital admissions.

#### 2.1.4 Analysis

First, we conducted descriptive analyses for the study population. Second, we performed collinearity diagnostics to test for collinearity among the four social needs variables and covariate measures. The results of the analysis found no collinearity and all social needs and covariate measures were included in two logistic regression models. In the first model, each social need variable was analyzed as a dichotomous measure: yes vs. no (ref. group); while in the second model, the count of social needs was analyzed: 1, 2, 3 or more vs. 0 needs (ref. group). All statistical analyses were conducted using SAS Version 9.4.

## 3 Results

### 3.1 Study characteristics

Nearly 60% of the population was between age 18 and 64, 60.4% were female, and about 80.1% were White (Table 1). Nearly 80% of the population lived in neighborhoods with ADI scores representative of moderate or lower area deprivation, while 37.3% had a CCI score of 1 or higher. Regarding prior health care utilization in the year prior to social needs assessment, 64.9% had 1 or more dental visits, 57.9% had 1 or more primary care visits, 22.4% had 1 or more ED visits and 9.1% had 1 or more hospital admissions.

**Table 1:** Population characteristics.

Characteristic	Total *N* = 80,273 *N* (%)
**Sociodemographic Characteristics**	
Age, *N* (%)	
18–44	24,453 (30.5%)
45–64	22,953 (28.6%)
≥65	32,857 (40.9%)
Mean +/- SD	55.9 +/- 18.3
Sex, *N* (%)	
Female (vs. male)	48,463 (60.4%)
Race/Ethnicity, *N* (%)	
Asian/Asian-American	3,914 (4.9%)
Black/African-American	2,323 (2.9%)
Hawaiian/Pacific Islander	279 (0.4%)
Hispanic/Latino	5,563 (6.9%)
Native American/Alaskan Native	232 (0.3%)
White	64,316 (80.1%)
More than one race/Other race	3,646 (4.5%)
Area Deprivation Index (ADI), *N* (%)	
Least deprivation (1–3)	32,270 (40.2%)
Moderate deprivation (4–6)	30,705 (38.3%)
Highest deprivation (7–10)	17,298 (21.6%)
**Comorbidities and Prior Health Care Utilization**	
Charlson Comorbidity Index, *N* (%)	
0	50,306 (62.7%)
1	12,199 (15.2%)
2+	17,768 (22.1%)
Utilization Characteristics (previous 12 months)	
+1 dental visits	52,135 (64.9%)
+1 primary care visits	46,489 (57.9%)
+1 ED visits	17,948 (22.4%)
+1 hospital admissions	7,266 (9.1%)

### 3.2 Type and number of social needs

Approximately 18% of patients reported one or more social needs, while 3.9% reported three or more needs (Table 2). The most common social need was financial strain (12.8%), followed by food insecurity (9.6%), housing instability (5.6%) and transportation needs (4.0%).

**Table 2:** Description of social needs.

Individual social needs	*N* (%)
Financial Strain	10,293 (12.8%)
Food Insecurity	7,701 (9.6%)
Housing Instability	4,504 (5.6%)
Transportation Needs	3,186 (4.0%)
**Count of Needs**	***N* (%)**
None	65,967 (82.2%)
One	6,833 (8.5%)
Two	4,308 (5.4%)
Three or more needs	3,165 (3.9%)
Total needs (mean +/- SD)	0.3 +/- 0.9

### 3.3 Logistic regression results: association of individual social needs with dental care utilization

Financial strain (OR = 0.82, 95% CI = 0.77–0.88), food insecurity (OR = 0.87, 95% CI = 0.81–0.93) and transportation needs (OR = 0.81, 95% CI = 0.74–0.88) were each associated with lower odds of having one or more dental visits in the year following social needs assessment (Table 3).

**Table 3:** Logistic regression results: association of individual social needs with dental utilization in 12 months post social needs assessment.

Individual social need	OR	95% CI	*p*-value
Financial strain	**0**.**82**	**0.77**–**0.88**	**<0**.**0001**
Food insecurity	**0**.**87**	**0.81**–**0.93**	**0**.**0001**
Housing instability	0.94	0.87–1.02	NS*
Transportation needs	**0**.**81**	**0.74**–**0.88**	**<0**.**0001**

Logistic regression model adjusted for age, sex, race/ethnicity, ADI, CCI, prior dental utilization, prior primary care utilization, prior ED utilization and prior hospital admissions.

*Not statistically significant at the *p* < 0.05 level.

Bolded text indicates statistically significant association between social needs and dental utilization.

### 3.4 Logistic regression results: association of count of individual social needs with dental care utilization in 12 months post social needs assessment

Compared to having no social needs, having one social need (OR = 0.77, 95% CI = 0.73–0.82), two social needs (OR = 0.74, 95% CI = 0.69–0.79) and three or more social needs (OR = 0.63, 95% CI = 0.58–0.69) were all strongly associated with lower odds of having any dental visit (Table 4). Each additional social need lowered the odds of dental utilization in the following year.

**Table 4:** Logistic regression results: association of count of individual social needs with dental utilization in 12 months post social needs assessment.

Count of social needs	OR	95% CI	*p*-value
None	**1**.**00**	**NA**	**NA**
One	**0**.**77**	**0.73–0.82**	**<0.0001**
Two	**0**.**74**	**0.69–0.79**	**<0.0001**
Three or more needs	**0**.**63**	**0.58–0.69**	**<0.0001**

Logistic regression model adjusted for age, sex, race/ethnicity, ADI, CCI, prior dental utilization, prior primary care utilization, prior ED utilization, and prior hospital admissions.

Bolded text indicates statistically significant association between social needs and dental utilization.

## 4 Discussion

We found that nearly 18% of our study population, age 18+ with dental and medical insurance coverage, reported one or more social needs, with about 10% reporting two or more social needs. The presence of financial strain, food insecurity and transportation needs were associated with lower odds of having any dental visits in the year after social needs assessment.

Our finding that financial strain was associated with lower use of dental care substantiates previous findings that affordability and cost serve as barriers to accessing dental care (10–12). Additionally, our study results support prior research indicating that food insecurity and transportation barriers are associated with lower use of dental care. Specifically, Wiener (2018) (14), Merchant (2024) (16) and Obeidat (2024) (15) found that food insecurity was associated with lower use of dental care services. Similarly, McKernan (2018) (19), Kim (2024) (18) and Obeidat (2024) (15) found that transportation barriers were associated with lower use of dental care. However, unlike previous research by Obeidat (2024) (15) and Yu (2025) (17) that found housing instability was associated with lower use of dental care, our study found no association between housing instability and dental care utilization.

Of critical importance, we also found a “dose-response” relationship between number of social needs and dental utilization, with each subsequent social need lowering the odds of a person having a dental visit. Consequently, multiple unmet social needs may create overlapping social barriers, draining a patient's time and resources, and forcing economic tradeoffs that may make routine or preventive dental care a lower priority.

Our study did have certain limitations. First, because the study is observational, we cannot establish a causal relationship between social needs and dental utilization. Second, results cannot be generalized outside of integrated health care systems. Third, because social needs data were self-reported, they may be subject to social desirability bias. Moreover, because the study population was mostly White, results cannot be generalized to populations that are more racially/ethnically diverse than the study population. Further, less information is known about whether the association of social needs and dental care utilization is similar in non-White patient populations. Last, results may not be applicable to non-insured populations.

These limitations notwithstanding, our study results have implications for policy and clinical practice. Although the associations of social needs with dental utilization are not causal, our results suggest that dental patients with financial strain, food insecurity and transportation needs may benefit from possible interventions to increase use of dental care. Possible interventions could include outreaching patients via a community health navigator to address social health needs such as, for example, financial strain. Community health navigators can help such patients apply for such services as medical financial assistance to reduce financial barriers to access needed dental services. Last, further research is needed to evaluate the effectiveness of interventions to target social needs and evaluate whether resolution of these needs is associated with increases in dental utilization.

## Data Availability

The data analyzed in this study is subject to the following licenses/restrictions: Data is proprietary and not publicly available. Further queries should be directed to david.m.mosen@kpchr.org.

## Appendix A

Appendix A Description of Social Needs.

**Table:**

Measure	Reponses below indicate “yes” response for social need
Financial strain (1 item)	Ability to pay for basics such as food, housing, medical care, and heating rated as “very hard,” “hard” or “somewhat hard”.
Food insecurity (2 items)	Rated the following as “sometimes true” or “often true”: In the past 12 months, 1) worried food would run out before more could be bought or 2) worried food bought would not last and did not have money to get more.
Housing instability (3 items)	In the past 12 months (including now), any of the following three conditions occurred: 1) unable to pay the mortgage or rent on time, 2) lived in 3 or more places or 3) did not have a steady place to sleep or slept in a shelter.
Transportation needs (2 items)	In the past 12 months, lack of transportation: 1) kept respondent from medical appointments or from getting medications or 2) kept respondent from meetings, work, or from getting things needed for daily living.

## References

[1] Taylor KK Neiman PU Bonner S Ranganathan K Tipirneni R Scott JW. Unmet social health needs as a driver of inequitable outcomes after surgery: a cross-sectional analysis of the national health interview survey. Ann Surg. (2023) 278(2):193–200. 10.1097/sla.0000000000005689 36017938 PMC10122453

[2] United States Department of Health and Human Services OoDPaHP. Social Determinants of Health - Healthy People 2030 (n.d.). Available online at: https://odphp.health.gov/healthypeople/priority-areas/social-determinants-health (Accessed April 16, 2026).

[3] Heller CG Rehm CD Parsons AH Chambers EC Hollingsworth NH Fiori KP. The association between social needs and chronic conditions in a large, urban primary care population. Prev Med. (2021) 153:106752. 10.1016/j.ypmed.2021.106752 34348133 PMC8595547

[4] Vrtikapa K Hoque Urmy F Hoque F. Social determinants of health: the impact of this overlooked vital sign. J Brown Hosp Med. (2025) 4(3):138072. 10.56305/001c.138072 40612083 PMC12224330

[5] Fiori KP Heller CG Rehm CD Parsons A Flattau A Braganza S. Unmet social needs and no-show visits in primary care in a US northeastern urban health system, 2018–2019. Am J Public Health. (2020) 110(S2):S242–50. 10.2105/ajph.2020.305717 32663075 PMC7362703

[6] Poleshuck E Possemato K Johnson EM Cohen AJ Fogarty CT Funderburk JS. Leveraging integrated primary care to address patients’ and families’ unmet social needs: aligning practice with National Academy of Sciences, Engineering and Medicine recommendations. J Am Board Fam Med. (2022) 35(1):185–9. 10.3122/jabfm.2022.01.210287 35039426 PMC12013516

[7] Mohd Khairuddin AN Bogale B Kang J Gallagher JE. Impact of dental visiting patterns on oral health: a systematic review of longitudinal studies. BDJ Open. (2024) 10(1):18. 10.1038/s41405-024-00195-7 38448428 PMC10917741

[8] Fu D Shu X Zhou GM Liao G Zou L. Connection between oral health and chronic diseases. Med Comm. (2025) 6(1):e70052. 10.1002/mco2.70052 PMC11731113 39811802

[9] D'Aiuto F Suvan J Siripaiboonpong N Gatzoulis MAD Aiuto F. The root of the matter: linking oral health to chronic diseases prevention. Int J Cardiol Congenit Heart Dis. (2025) 19:100574. 10.1016/j.ijcchd.2025.100574 40066344 PMC11891740

[10] Vujicic M Buchmueller T Klein R. Dental care presents the highest level of financial barriers, compared to other types of health care services. Health Aff (Millwood). (2016) 35(12):2176–82. 10.1377/hlthaff.2016.0800 27920304

[11] Flynn B Starkel Weninger R Zaborowski M Vujicic M. Barriers to Dental Care among Adult Medicaid Beneficiaries Chicago, IL (2024). Available online at: https://www.ada.org/-/media/project/ada-organization/ada/ada-org/files/resources/research/hpi/barriers_Medicaid_participation_utilization.pdf (Accessed April 16, 2026).

[12] The Commonwealth Fund. Medicare Beneficiaries with Dental Insurance Face Care Barriers (2025). Available online at: https://www.commonwealthfund.org/blog/2025/many-medicare-beneficiaries-dental-insurance-face-financial-barriers-care (Accessed June 29, 2026).

[13] Velez M Buto PT Pederson AM Weuve J Murchland AR Wang J. Associations of unmet dental care needs due to cost with incident cardiovascular disease and dementia: a prospective study in the all of US cohort. J Gerontol A Biol Sci Med Sci. (2026) 81(4):glag023. 10.1093/gerona/glag023 41678708 PMC13016879

[14] Wiener RC Sambamoorthi U Shen C Alwhaibi M Findley P. Food security and unmet dental care needs in adults in the United States. J Dent Hyg. (2018) 92(3):14–22. 29976789 PMC6059372

[15] Obeidat R Heaton LJ Tranby EPO Malley J Timothé P. Social determinants of health linked with oral health in a representative sample of U.S. adults. BMC Oral Health. (2024) 24(1):1518. 10.1186/s12903-024-05257-8 39707273 PMC11660569

[16] Merchant AT Fallahi A Huda A Lohman M. Food insecurity and oral health in older adults. Front Oral Health. (2024) 5:1400591. 10.3389/froh.2024.1400591 39512559 PMC11540812

[17] Yu YH. Access to oral health care and its social determinants across the lifespan in the United States. Front Oral Health. (2025) 6:1619983. 10.3389/froh.2025.1619983 41019453 PMC12460411

[18] Kim J Roy I Martinez-Mier EA Shukla A Weir P. Impact of lack of transportation on access to dental care. Heliyon. (2024) 10(23):e40657. 10.1016/j.heliyon.2024.e40657 39698077 PMC11652834

[19] McKernan SC Reynolds JC Ingleshwar A Pooley M Kuthy RA Damiano PC. Transportation barriers and use of dental services among Medicaid-insured adults. JDR Clin Trans Res. (2018) 3(1):101–8. 10.1177/2380084417714795 30938652

[20] Mosen DM Banegas MP Dickerson JF Fellows JL Brooks NB Pihlstrom DJ. Examining the association of medical-dental integration with closure of medical care gaps among the elderly population. J Am Dent Assoc. (2021) 152(4):302–8. 10.1016/j.adaj.2020.12.010 33775288 PMC9573779

[21] Mosen DM Banegas MP Dickerson JF Fellows JL Pihlstrom DJ Kershah HM. Evaluating the effectiveness of medical-dental integration to close preventive and disease management care gaps. Front Dent Med. (2021) 2:670012. 10.3389/fdmed.2021.670012 36213339 PMC9536421

[22] Hahn EA DeWalt DA Bode RK Garcia SF DeVellis RF Correia H. New English and Spanish social health measures will facilitate evaluating health determinants. Health Psychol. (2014) 33(5):490–9. 10.1037/hea0000055 24447188 PMC4159098

[23] Cella D Riley W Stone A Rothrock N Reeve B Yount S. The patient-reported outcomes measurement information system (PROMIS) developed and tested its first wave of adult self-reported health outcome item banks: 2005–2008. J Clin Epidemiol. (2010) 63(11):1179–94. 10.1016/j.jclinepi.2010.04.011 20685078 PMC2965562

[24] Posner BM Jette AM Smith KW Miller DR. Nutrition and health risks in the elderly: the nutrition screening initiative. Am J Public Health. (1993) 83(7):972–8. 10.2105/AJPH.83.7.972 8328619 PMC1694757

[25] Puterman E Haritatos J Adler NE Sidney S Schwartz JE Epel ES. Indirect effect of financial strain on daily cortisol output through daily negative to positive affect Index in the coronary artery risk development in young adults study. Psychoneuroendocrinology. (2013) 38(12):2883–9. 10.1016/j.psyneuen.2013.07.016 23969421 PMC3844074

[26] National Association of Community Health Centers. Association of Asian Pacific Community Health Organizations, Oregon Primary Care Association, and Institute for Alternative Futures. The Protocol for Responding to and Assessing Patients’ Assets, Risks, and Experiences (Prapare) (2023). Available online at: https://prapare.org/ (Accessed May 26, 2023).

[27] Childrens Health Watch. Housing Instability: A New Screen for Adverse Health Issues for Caregivers and Children (2018). Available online at: https://childrenshealthwatch.org/housing-instability-a-new-screen-for-adverse-health-issues-for-caregivers-and-children/ (Accessed May 26, 2023).

[28] Hall MH Matthews KA Kravitz HM Gold EB Buysse DJ Bromberger JT. Race and financial strain are independent correlates of sleep in midlife women: the swan sleep study. Sleep. (2009) 32(1):73–82. 19189781 PMC2625326

[29] Hager ER Quigg AM Black MM Coleman SM Heeren T Rose-Jacobs R. Development and validity of a 2-item screen to identify families at risk for food insecurity. Pediatrics. (2010) 126(1):e26–32. 10.1542/peds.2009-3146 20595453

[30] Mosen DM Fitzpatrick SL Keast EM Dickerson JF Ertz-Berger BL Banegas MP. Association of social needs with diabetes outcomes in an older population. J Am Board Fam Med. (2025) 38(1):125–32. 10.3122/jabfm.2024.240139R2 40268320 PMC12096382

[31] Mosen DM Banegas MP Keast EM Dickerson JF. Examining the association of social needs with future health care utilization in an older adult population: which needs are most important? Popul Health Manag. (2023) 26(6):413–9. 10.1089/pop.2023.0171 37943589 PMC10698796

[32] Tuliani TA Shenoy M Parikh M Jutzy K Hilliard A. Impact of area deprivation index on coronary stent utilization in a Medicare nationwide cohort. Popul Health Manag. (2017) 20(4):329–34. 10.1089/pop.2016.0086 28106520

[33] Kieszak SM Flanders WD Kosinski AS Shipp CC Karp H. A comparison of the charlson comorbidity index derived from medical record data and administrative billing data. J Clin Epidemiol. (1999) 52(2):137–42. 10.1016/s0895-4356(98)00154-1 10201654

[34] Deyo RA Cherkin DC Ciol MA. Adapting a clinical comorbidity index for use with icd-9-cm administrative databases. J Clin Epidemiol. (1992) 45(6):613–9. 10.1016/0895-4356(92)90133-8 1607900

